# Damage Monitoring of Steel Bars Based on Torsional Guided Waves

**DOI:** 10.3390/s24072047

**Published:** 2024-03-22

**Authors:** Zhupeng Zheng, Zihao Zhang, Cheng Huang

**Affiliations:** 1 Department of Civil Engineering, Xiamen University, Xiamen 361005, China; shallowsing95@gmail.com; 2 Shenzhen Research Institute, Xiamen University, Shenzhen 518087, China; 3 Xiamen R&B Baicheng Construction Investment Co., Ltd., Xiamen 361009, China; bcbgs1403@126.com

**Keywords:** torsional guided wave, damage monitoring, reflectivity, sensitivity analysis

## Abstract

Ultrasonic guided waves represent a new development in the field of non-destructive testing. Longitudinal guided waves are mostly used to monitor the damage of steel bars, but the received signal is usually degraded and noisy owing to its dispersive propagation and multimodal behavior, making its implementation and location challenging. The torsional mode of T (0, 1) is not dispersive in the propagation of a steel bar and only produces circumferential displacement. It was chosen, in this study, to conduct guided wave-based damage monitoring on steel bars to reduce the signal processing complexity. The defects of steel bars, including circular surface defects, internal defects, and uniform damage defects, were thoroughly investigated, respectively, using numerical simulation. The waves were excited and received using the pitch-and-catch technique and the collected monitoring signals were processed using Hilbert transformation to highlight the amplitude and time-of-flight values of the wave signals, which were used for defect identification. In this paper, the reflectivity of guided waves is compared between torsional waves and longitudinal waves, in each case. The impact of defect size changes on damage monitoring is studied and the sensitivity of both the wave frequency and the wave mode (L and T) is also discussed. The results show that the monitoring method based on the torsional wave T (0, 1) is more sensitive to surface defects than the conventional method based on longitudinal waves. The reflectivity of the torsional wave T (0, 1) can be twice that of the longitudinal wave L (0, 1) when the depth of the defect in the circumferential grooves is less than 50% of the diameter of the steel bar. It is more sensitive to shallow surface defects within half of the bar’s radius, and it can also effectively identify defects under the conditions of the uniform damage defects of steel bars, even when the measurements are heavily noise-polluted. This proves the superiority of the torsional guided wave T (0, 1) in defect monitoring and provides a theoretical basis for the application of the torsional guided wave T (0, 1) in actual monitoring.

## 1. Introduction

Since the 1990s, monitoring technologies based on ultrasonic guided waves have been gradually explored and applied in the field of non-destructive monitoring. Compared with traditional ultrasonic monitoring technology, this new technology using ultrasonic guided waves to monitor materials and structures in solid media is faster, more sensitive, and more economical. This is a newly developed subject in the field of non-destructive monitoring [1]. In the past 30 years, the literature on ultrasonic guided waves has increased in amount significantly and the discussed research fields have covered pipes, solid cylinders, composite plates, and more [2,3]. In the petrochemical and transportation industries, cylindrical constructions such as rods and pipes are commonly used. Because of the hard-working conditions in these industries, these structures could decay and constitute a safety risk. As a result, cylindrical structures must be inspected on a frequent basis to ensure their safe functioning [4,5]. For evaluating cylindrical structures, ultrasonic guided wave technology has been used [6,7]. This non-destructive testing technology is gaining popularity because it enables the possibility of long-distance examinations from a single probe location, the inspection of unreachable areas, and great efficiency.

Many scholars have conducted relevant studies on the damage monitoring of bare steel bars. Previous research has indicated that longitudinal guided waves are mostly used to monitor the damage of steel bars. For example, Ervin et al. [8] used the fundamental longitudinal mode L (0, 1) and the L (0, 9) mode in both low and high frequency ranges, respectively, to monitor damage in reinforced mortar specimens undergoing accelerated uniform corrosion and concluded that the L (0, 1) mode was not detected until after corrosion had been initiated, because it was appreciably attenuated for the particular specimen size used. In addition, the L (0, 9) mode was relatively insensitive to the surrounding interface conditions at high frequencies. Amjad [9] successfully identified the diameter of the steel bar after corrosion by applying the principle of reducing the diameter of the steel bar to change the wave velocity of the L (0, 1) mode. Alireza [10] studied the change in diameter in the corrosion process of steel strands by exciting the L (0, 1) mode and used the change in group velocity to identify the diameter of the corroded strands. As longitudinal guided waves are frequently employed for monitoring steel bar damage, the received signal is usually degraded and noisy owing to its dispersive propagation and multimodal behavior, making its implementation and location challenging [11].

Since the use of torsional-mode guided waves for damage monitoring has been carried out by many researchers, there are few studies detailing sensitivity analyses on steel bars. The application and advancement of torsional guided waves has lagged behind that of longitudinal guided waves due to their more complex excitation methods in structures [12]. The torsional mode T (0, 1) is non-dispersive in the propagation of the steel bar and only produces circumferential displacement, which has its advantages in the monitoring of circumferential surface defects. Some researchers have recently employed PZT to excite a fully axisymmetric torsional wave. Peking University’s Li et al. [13,14] effectively produced entirely axis-symmetrical torsional-mode waves in aluminum tubes, using 24-face shear d24 PZT elements excited at 150 kHz. This has been, so far, mostly used for the structural health monitoring of pipes [14,15]. Yeung and Ng [16] investigated the effect of crack size on the scattering and mode-converted guided waves of T (0, 1) in pipes using a time-domain spectral finite-element method (SFEM), which was found to be able to predict torsional guided wave propagation, scattering, and mode conversion accurately. However, the SFEM is not broad enough and its computational format also lacks universality. The phenomenon of guided wave mixing in pipes, in both the presence and absence of embedded soil, has also been investigated numerically by Ng and Yeung et al. [17]. They developed a three-dimensional (3D) finite-element (FE) model incorporating a strain energy function to analyze the wave-mixing phenomena in pipes embedded in the soil. Two torsional guided waves interacting with each other were simulated and the results showed that the amplitude in the frequency domain exhibited a sharp drop, due to the energy leakage in the existence of soil medium at fundamental frequencies and second and combinational harmonics. Few studies have evaluated the defect detection of steel bars using a comparison of the reflectivity via the torsional mode T (0, 1). Therefore, this paper aims to use the torsional mode T (0, 1) to monitor the damage of steel bars and to provide a theoretical basis for practical applications. Three cases of defects, including circular surface defects, internal defects, and uniform damage defects, are investigated, respectively. Sensitivity analysis is carried out through the reflectivity of guided waves and the results are compared with the method based on longitudinal guided waves in each case. The primary innovations presented in this paper include the introduction of a novel method for monitoring steel bar damage using torsional guided waves and a comparative analysis of this method’s sensitivity against the conventional approach, offering advancements in the field.

The remainder of this paper is structured as follows: Section 2 describes the fundamental properties of the torsional-mode guided wave of T (0, 1). Section 3 presents the numerical simulation of defect identification in steel bars based on T (0, 1) for three cases, respectively, which demonstrates the effectiveness of the T (0, 1)-based method, while Section 4 offers the concluding remarks.

## 2. Fundamental Properties of Torsional-Mode Guided Wave T (0, 1) 

**Figure 1:**
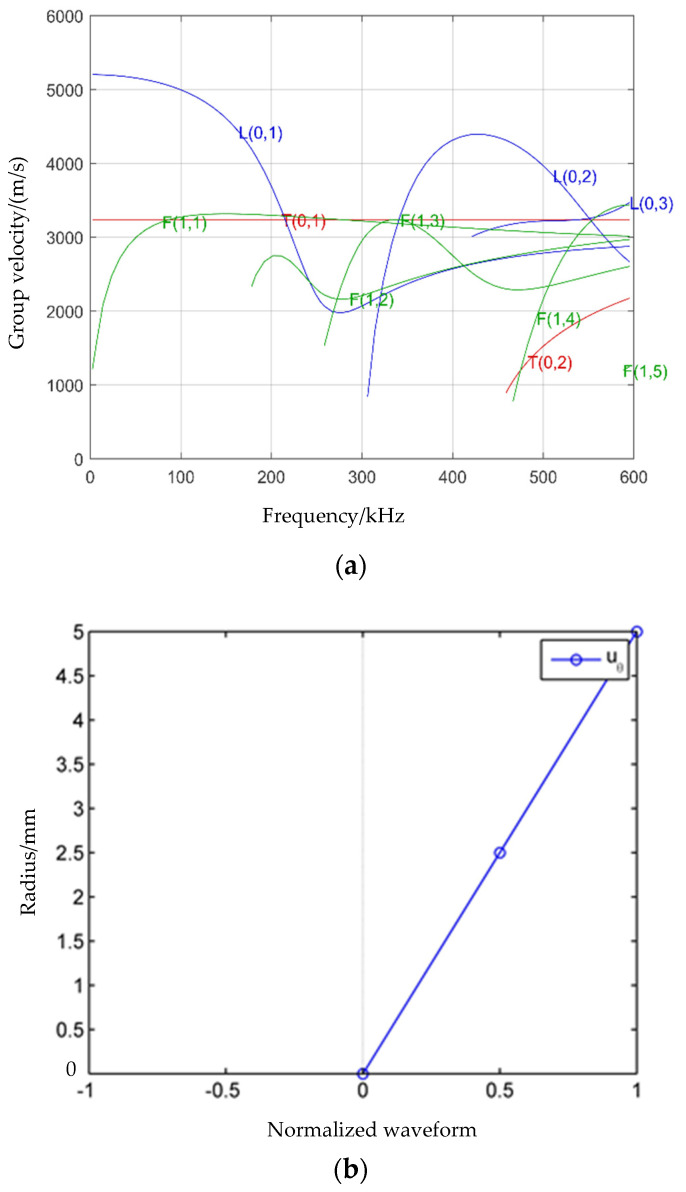
(**a**) Group velocity dispersive curves in a steel bar with a diameter of 10 mm. (**b**) Structural displacement diagram of T (0, 1).

The characteristics of guided waves have been explored in previous research by the authors of this paper [18,19,20,21]. As shown in Figure 1, the dispersive curves of guided waves for a steel bar with a diameter of 10 mm, and the wave structure diagram of the torsional wave T (0, 1) of a steel bar at 50 kHz, are plotted using the open-source program PCDISP and the GUIGUW 2.2 software, respectively. These tools allow determination of the group velocity and phase velocity of distinct modes at any frequency, as well as the displacement component of the guided mode at a certain frequency [22]. Consequently, the advantages of defect monitoring using the torsional-mode guided wave T (0, 1) become evident.

Figure 1a illustrates that the torsional mode of T (0, 1) exhibits non-dispersive behavior, indicating that the velocity remains constant regardless of frequency variations. Utilizing the torsional mode T (0, 1) as a monitoring technology results in a single excited guided wave due to its non-dispersive nature. This characteristic leads to reduced errors and enhanced precision in defect position identification compared to the monitoring method employing the L (0, 1) mode. As depicted in Figure 1b, the torsional mode exclusively displays circumferential displacement, with the maximum displacement occurring on the surface. This feature appears to make it more responsive to surface defect monitoring in steel bars than the longitudinal guided wave. Building upon these characteristics, this study will delve into the damage monitoring of steel bars using the torsional mode, with an accompanying analysis of sensitivity in comparison to the longitudinal-mode wave.

## 3. Numerical Simulation of Defect Identification in Steel Bars Based on T (0, 1)

### 3.1. Case I: Circular Surface Defects

#### 3.1.1. Modeling and Signal Processing

**Figure 2:**
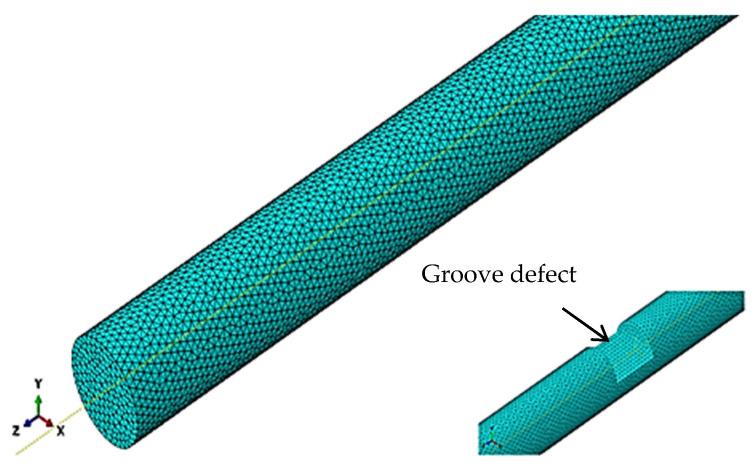
Modeling and groove defect diagram of a steel bar in ABAQUS.

As shown in the numerical modeling diagram in Figure 2, a 1 m long steel bar model with a diameter of 10 mm was constructed using ABAQUS with the following material parameters: elastic modulus E = 208.3 GPa, density ρ = 7700 kg/m^3^, and Poisson’s ratio μ = 0.29. A groove defect was introduced in the middle of the steel bar, with a defect axial length of 10 mm and a defect depth ranging from 1 mm to 5 mm. The windowed five-peak-wave Hanning signal was chosen to excite the guided waves at the left end of the steel bar, with an excitation center frequency of 100 kHz, and the signal was received at a quarter to the left.

The time step and spatial size are two important parameters that need to be controlled in numerical simulations, and improper settings can lead to non-convergence of the calculation. Regarding time steps, the smaller the step size, the higher the calculation accuracy, but this also increases the calculation time. This numerical simulation study utilizes ABAQUS’s automated integration step size to ensure computation convergence.

**Figure 3:**
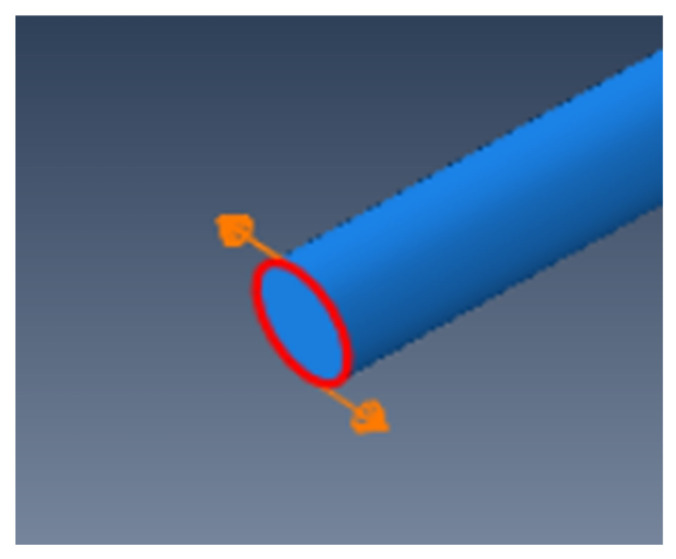
Schematic diagram of guided wave excitation of T (0, 1).

For grid partitioning, relevant studies have shown [23] that when a wavelength range contains 10 to 20 unit nodes, the accuracy of a numerical simulation analysis can be guaranteed. Therefore, the unit length Δ*l* is set to *λ*/20~*λ*/10, where *λ* is the wavelength. The formula for this is as follows:

(1) $$\Delta l\leq \left(\frac{\lambda }{20}-\frac{\lambda }{10}\right)=\left(\frac{C_{T}}{20f_{\max}}-\frac{C_{T}}{10f_{\max}}\right)$$

where *C_T_* is the velocity of the transverse wave, which is 3238 m/s in this example. *f_max_* is the maximum frequency involved in the study, in which only frequencies below 200 kHz are considered. After calculation, the size range of the grid is 0.81–1.6 mm, so 1 mm is chosen as the grid size for this example. The eight-node hexahedral element C3D8R is used for simulation. Only a circumferential displacement load needs to be applied at the end of the steel bar to excite the T (0, 1)-mode guided wave, as only axial displacement occurs. The guided wave excitation diagram is shown in Figure 3.

**Figure 4:**
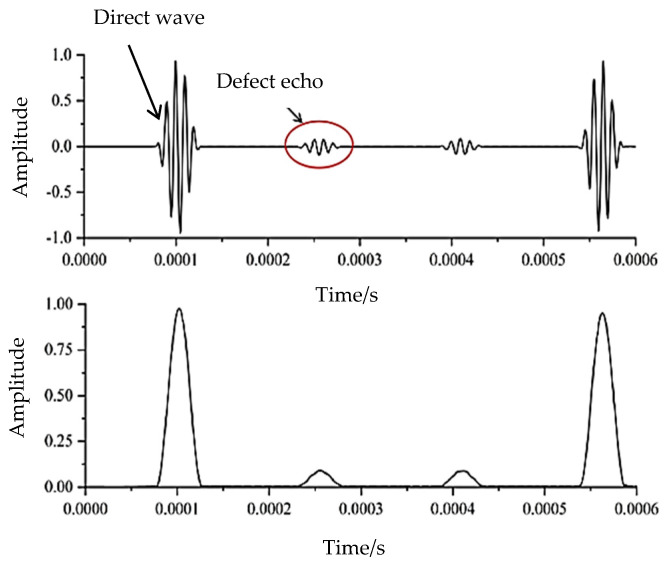
Received signal and its Hilbert transform.

When the defect depth is 1 mm, the signal’s time history curve obtained in the numerical simulation is successfully collected and processed via Hilbert transformation [24], as shown in Figure 4. The amplitude of a guided wave signal and its corresponding time can be clearly and intuitively identified through Hilbert transformation processing, which maps the signal from one time domain to another through mathematical processing. This facilitates the highlighting of characteristic information and enables defect location and damage analysis. The existence of a defect echo can be clearly observed in the red circle in Figure 4, and the propagating path length calculated using the product of the peak corresponding time interval length and velocity after Hilbert transformation is 0.498 m, which is only 0.4% in error compared with the theoretical 0.5 m. This demonstrates that the numerical simulation experiment successfully excites the torsional guided waves and completes the defect monitoring.

#### 3.1.2. Comparison of Sensitivity to Defect Depth between Longitudinal and Torsional Waves

Through the analysis of the waveform structure of the longitudinal and torsional waves, it becomes evident that the displacement of the torsional guided wave mainly occurs on the surface, suggesting that it should be more sensitive to defect monitoring on the circumferential surface. This hypothesis will now be discussed and verified using another numerical simulation experiment. The defect model is set as shown in Figure 2, and the defect is monitored using both the longitudinal mode of L (0, 1) and the torsional mode of T (0, 1), respectively. The ratio of the signal amplitude of the defect echo to the direct wave after Hilbert transformation, i.e., the defect reflectivity, is taken as the test basis for this analysis.

**Figure 5:**
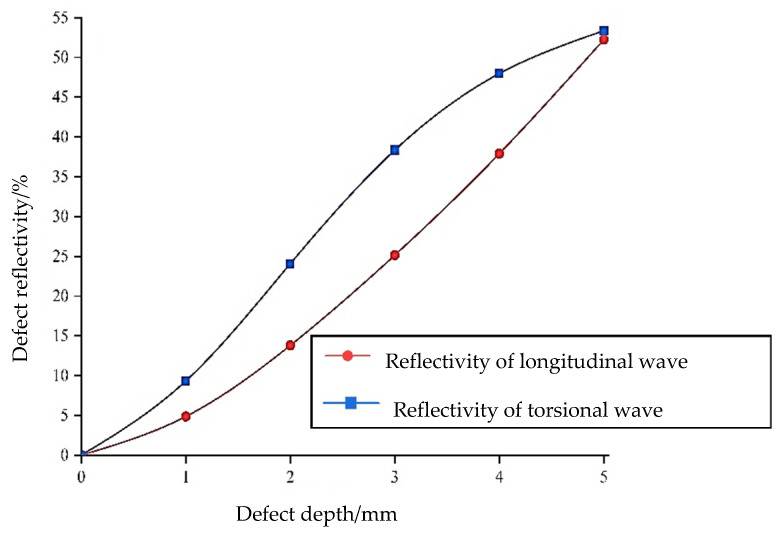
Comparison of sensitivity to defect depth between longitudinal and torsional waves.

From the results shown in Figure 5, it can be clearly observed that when the depth of the defect is less than 5 mm, which is 50% of the diameter of the steel bar, the torsional wave is more sensitive to defect monitoring than the longitudinal wave. Specifically, when the defect depth is as small as 2 mm, the reflectivity of the torsional wave is 24%, approximately twice that of the longitudinal wave at 13.8%. The assumption that the torsional wave is more sensitive to surface defects is well proved by these numerical simulation experiments.

**Figure 6:**
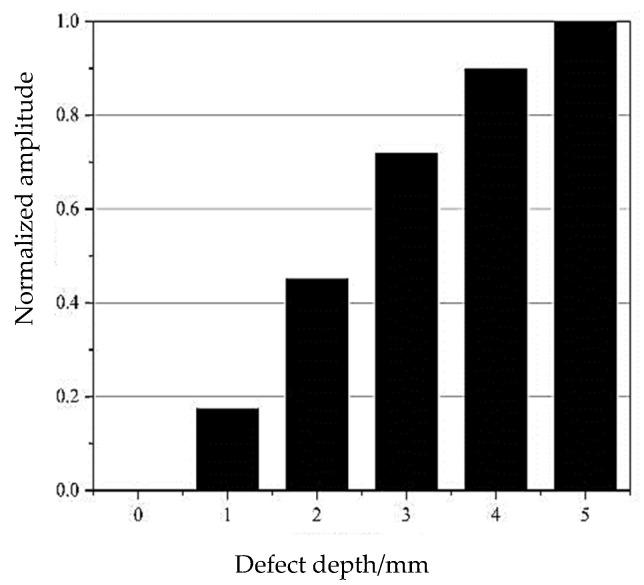
Normalized amplitude of the reflected echo.

In order to more intuitively demonstrate the influence of defect size on reflected echo amplitudes, the defect echo reflectivity is normalized, as shown in Figure 6. The figure illustrates that the greater the depth of defects, the higher the normalized amplitude. The normalized value can be used to quantitatively identify the damage defects.

#### 3.1.3. The Effect of Frequency on Defect Monitoring When Using Torsional Waves

**Figure 7:**
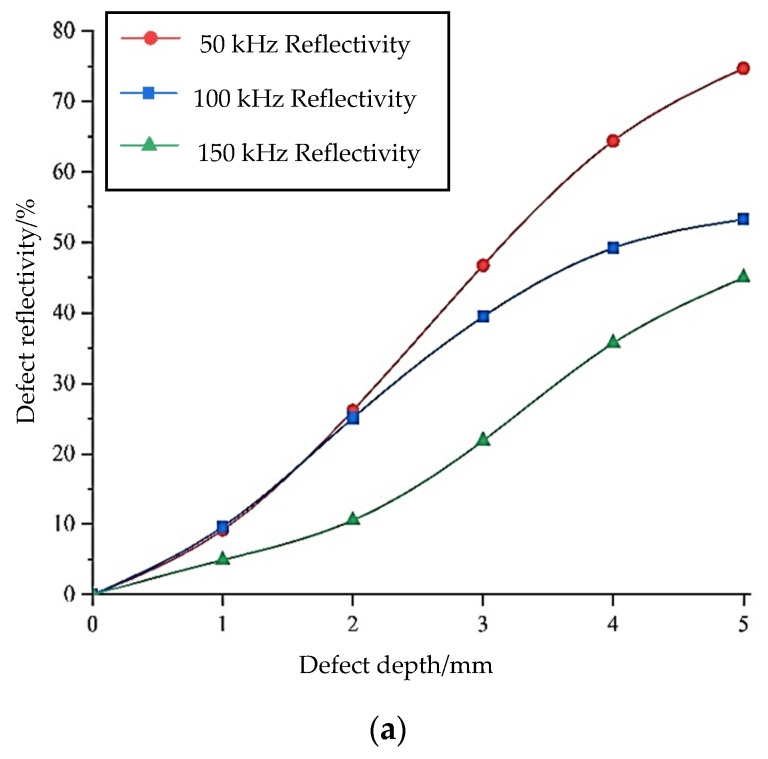

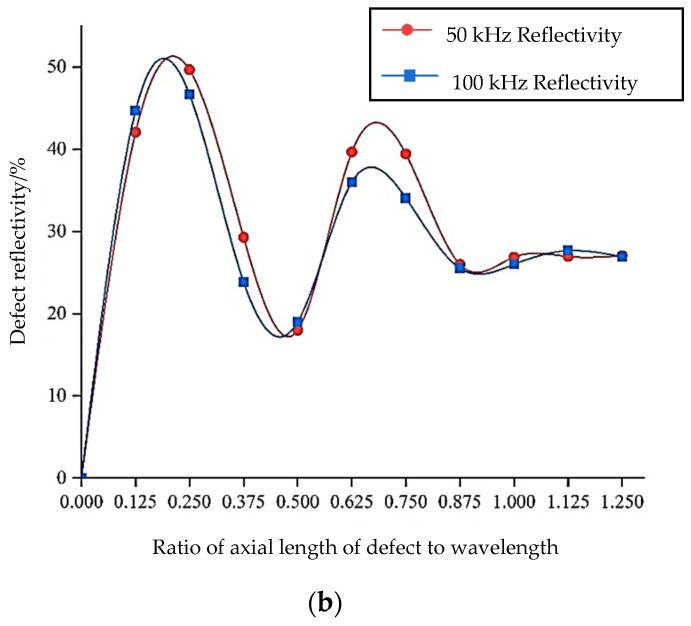
Influence of torsional waves on defect monitoring at different frequencies: (**a**) sensitivity to the depth of the defect; (**b**) sensitivity to the axial length of the defect.

After recognizing the advantages of torsional waves, the influence of different excitation frequencies on monitoring using torsional waves is investigated. As depicted in Figure 1a, it is evident that there are few types of guided wave modes when the frequency is below 200 kHz. Therefore, it is appropriate to test with frequencies lower than 200 kHz. The effects of torsional guided waves on changes in the depth of defects and axial defects of the steel bar at different frequencies are considered separately, and these results are shown in Figure 7.

As illustrated in Figure 7a, guided waves at low frequencies are more sensitive to defect monitoring compared to those at high frequencies. The reason for this is that when the center frequency of the excitation signal is low, its wavelength is large, and the attenuation is small during propagation. An analysis of the torsional wave for the monitoring of defects at various axial lengths is shown in Figure 7b. The observation reveals that, when the axial length of the defect changes, the signal changes periodically under the influence of wave interference. It reaches its maximum when the ratio of the axial defect length to the wavelength is one quarter and reaches its minimum when the ratio is half. It shows a stable trend when the ratio is more than one. The guided waves propagate along the axial direction of the component. When the defect length is equal to one-quarter of the wavelength and just touches the peak or valley of the guided wave, the amplitude of the wave is at its maximum, resulting in a maximum reflection coefficient. When the defect length is equal to three-quarters of the wavelength, it is also within the peak or valley of the guided wave, resulting in a second maximum value. When the defect length is equal to half the wavelength, this happens to be at the stationary point of the guided wave, with the smallest amplitude, resulting in a minimum reflection coefficient. These results are consistent with those from Wang and others [25,26] on the monitoring of axial length defects in steel bars and pipes. This shows that the torsional guided wave is less sensitive to axial defect length monitoring than defect depth monitoring. When the influence of different exciting frequencies on monitoring is analyzed, the results show that it is not greatly affected by frequencies.

### 3.2. Case II: Internal Defects

#### 3.2.1. Comparison of Sensitivity to Shallow Surface Defect Monitoring between Longitudinal and Torsional Waves

When the defect is located inside the steel bar, the monitoring’s sensitivity to the defect when using the longitudinal wave L (0, 1) and the torsional wave T (0, 1), respectively, can be analyzed as follows.

**Figure 8:**
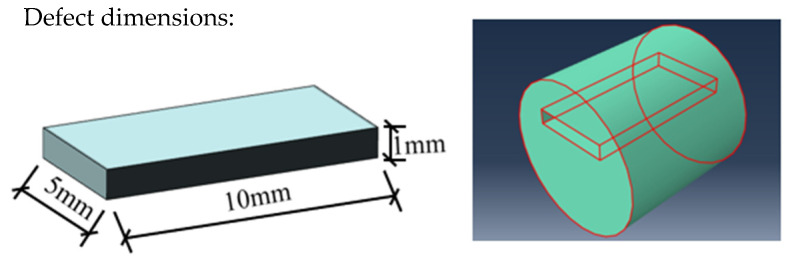
Diagram of an internal defect.

An internal rectangular defect is created with dimensions of 10 mm × 5 mm × 1 mm in length, width, and height, respectively, as shown in Figure 8.

**Figure 9:**
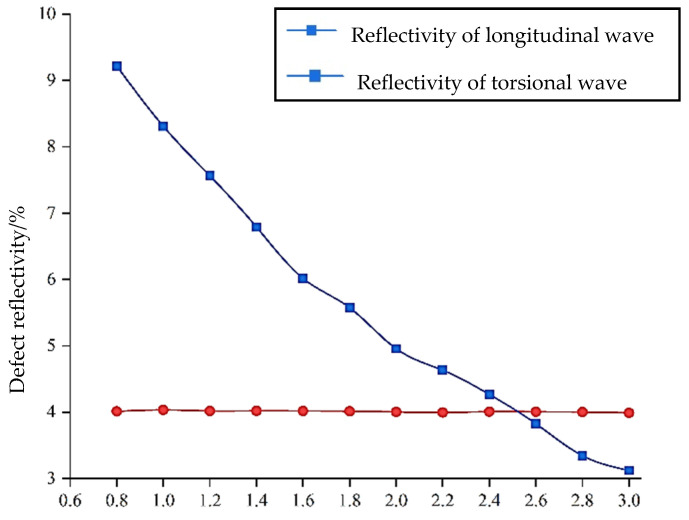
Comparison of sensitivity between longitudinal and torsional waves for defect monitoring on a shallow surface.

The defect depth is defined by the distance from the top surface of the defect to the outer surface of the element. In Case I, it is evident that the frequency of 50 kHz corresponds to the maximum reflectivity for various defect dimensions. Hence, the discussion in this case focuses on the signal at 50 kHz. The final result is shown in Figure 9, wherein the defect depth starts at 0.8 mm and increases in increments of 0.2 mm with the excitation frequency of 50 kHz.

It can be seen from Figure 9 that the result is well in line with the characteristics of the waveform structural diagram. The defect reflectivity of the longitudinal guided wave is basically maintained at about 4%, while the reflectivity of the torsional guided wave shows a linear change regulation, which gradually reduces from the initial 9.22% to 3.12%. Also notable in the figure is that the two data curves intersect at a defect depth of about 2.5 mm, indicating that when the defect is located on the shallow surface of the waveguide structure, the torsional wave is more sensitive to defect monitoring on the shallow surface than the longitudinal wave, and the sensitivity range is about half of the radius of the steel bar.

#### 3.2.2. Influence of Three-Dimensional Changes in Shallow Surface Defects on Monitoring

**Figure 10:**
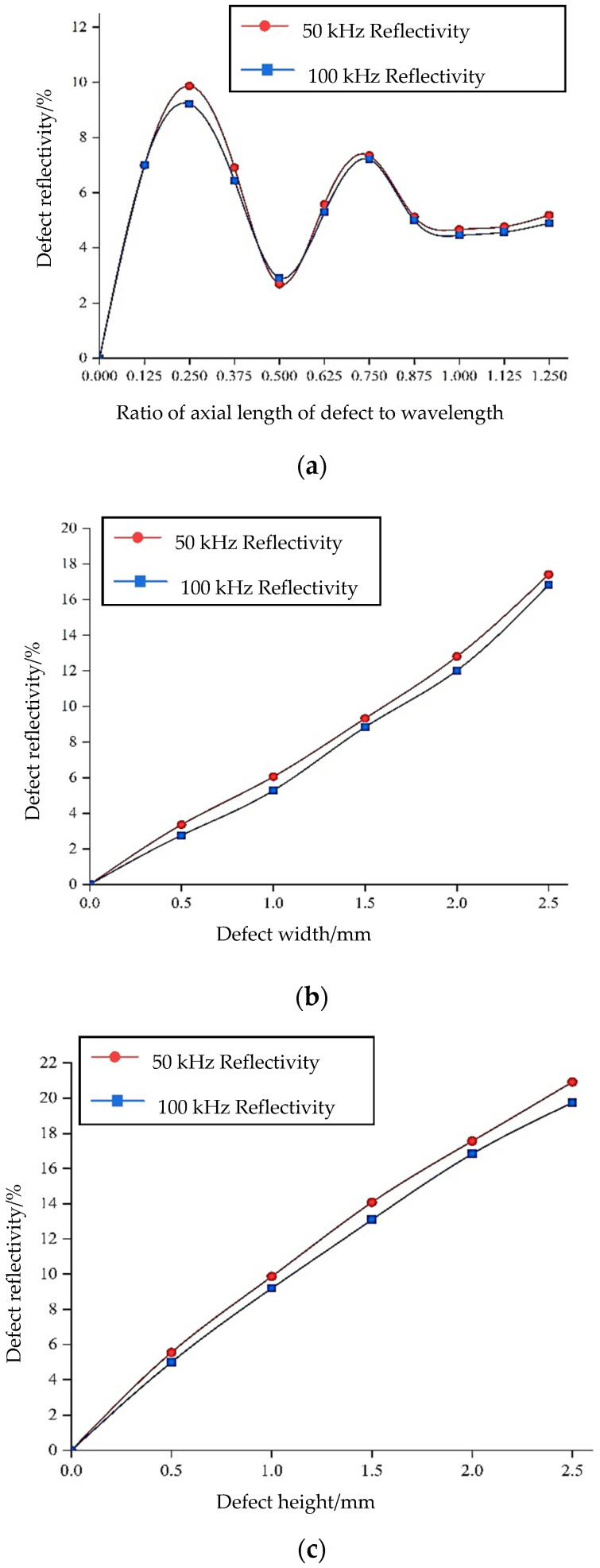
Impacts of three-dimensional changes in defects on monitoring: (**a**) the impact of changes in the axial length of defects on monitoring; (**b**) the impact of defect width changes on monitoring; (**c**) the impact of changes in defect height on monitoring.

When the defect is located inside the steel bar, the influence of three-dimensional changes in the defect’s length, width, and height on the monitoring can be analyzed as follows. In this numerical simulation experiment, only one of the dimensions is changed at a time, while the dimensions of the other two aspects remain unchanged. At the same time, considering the influence of frequency on monitoring, 50 kHz and 100 kHz are selected as the excitation center frequencies for each experiment. The final results are shown in Figure 10.

From these results, it is observed that when the size of the circular section changes (i.e., when the defect width and height change), the defect reflectivity increases linearly with this increase. Moreover, the difference in defect reflectivity under different frequencies is only about 1%, indicating that the frequency factor has little effect on the monitoring results. On the other hand, when the axial length of the defect changes, the defect reflectivity changes periodically with its increase, consistent with the change regulation of the defect on the outer surface of the circumference. Meanwhile, the frequency has little effect on changes in the axial dimension, and the defect reflectivity is basically the same under the two frequencies.

Demma et al. conducted a quantitative analysis of T (0, 1)-mode reflection from pipe faults within the 10–300 kHz frequency range [27]. Notches with different axial extents and crack-like flaws with zero axial extent were both taken into consideration. Their findings demonstrate that the reflection coefficient from axisymmetric fissures grows with the frequency at any given depth and increases monotonically with depth at all frequencies. With non-axisymmetric cracks, the reflection coefficient is a roughly linear function of the circumferential extent of the defect. Xu et al. [28] studied the locating method of longitudinal cracks in pipes using ultrasonic guided wave T (0, 1). Their results show that the maximum circumferential reflection coefficient appears in the circumferential position corresponding to the crack; the echo reflection coefficient first increases and then decreases with the crack length growth. In this way, the axial and circumferential locations of longitudinal cracks via the T (0, 1) torsion wave can be obtained. Their findings align with the conclusions drawn from the numerical simulations presented in this paper.

### 3.3. Case III: Uniform Damage Defects

#### 3.3.1. Comparison of Sensitivity to Uniform Damage Defect Monitoring between Longitudinal and Torsional Waves

**Figure 11:**
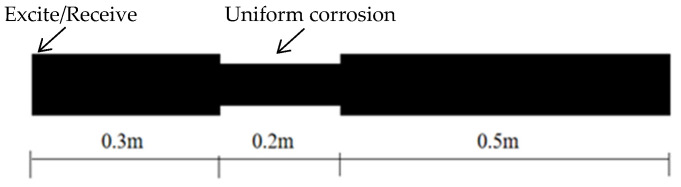
Schematic diagram of the numerical simulation of a uniform damage defect on a steel bar.

**Figure 12:**
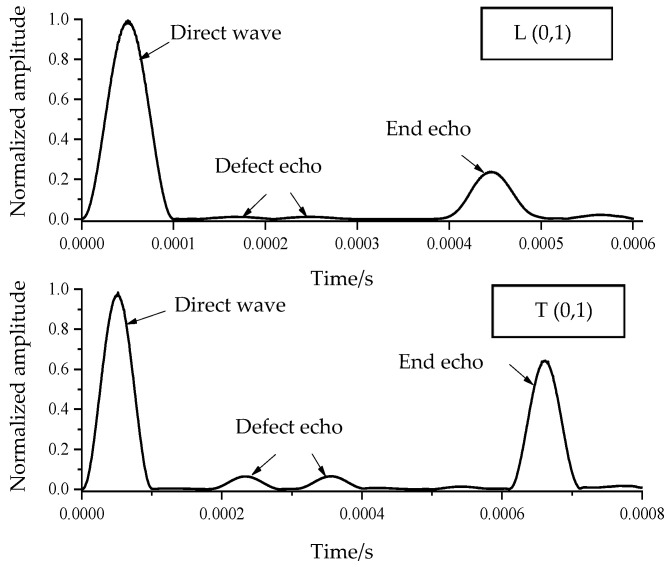
The received signal time history from longitudinal and torsional waves via the Hilbert transform.

A steel bar with a diameter of 20 mm and a length of 1 m is modeled using ABAQUS. The numerical simulation diagram of the steel bar is shown in Figure 11, and the material parameters are the same as those described in the previous chapters. The diameter of the steel bar is reduced in the middle to simulate a uniform damage defect of the steel bar. The length of the uniform damage defect is 20 cm, and the defect depth is 0.5 mm. Torsional guided waves and longitudinal guided waves are, respectively, selected to excite and receive signals at the left end of the steel bar. The excitation frequency is 50 kHz, and the received signal is processed using the Hilbert transform. The received signals are shown in Figure 12.

Figure 12 clearly demonstrates that the defect echoes at the front and rear ends of the defect are more obvious when the torsional guided wave is applied, while it is difficult to identify the defect echoes when using the longitudinal wave. Upon comparing the echo data, it is found that the reflectivity of the torsional guided wave is about 5.5 times that of longitudinal guided wave, indicating the super performance of the torsional guided wave over the longitudinal guided wave. When a uniform defect depth of only 0.5 mm is also analyzed, i.e., the working condition of small defects, it shows that the torsional guided wave is also more sensitive to the detection of small defects than the longitudinal guided wave.

#### 3.3.2. Identification of Uniform Damage Defects on a Steel Bar

After concluding that the application of the torsional guided wave T (0,1) in defect monitoring is superior to that of the longitudinal wave, further analysis can be conducted regarding the positioning and quantitative assessment of uniform damage defects in the steel bar, as follows.

As shown in Table 1, four groups of different working conditions are established. The length of the steel bar is 1 m, with a diameter of 20 mm, and a defect depth of 2 mm. The defect length varies across the conditions, as indicated by the second number in each working condition. The first and third numbers in the working condition denote the lengths of non-destructive steel bars in the front and back sections, respectively. Upon observing the numerical simulation results in Table 1, it becomes evident that under the four working conditions, the torsional guided wave exhibits extremely high accuracy in the determining the starting position of the defect and the defect length. Furthermore, the maximum error is no more than 2%, which proves that the torsional guided wave monitoring method can be used to accurately determine the location of uniform damage defects in a steel bar. For reference, Kim and Park [29] found that there is a certain linear relationship between the length or depth of uniform corrosion and the reflection coefficient. The position of local corrosion and its conditions can be analyzed using the numerical simulation method. This finding aligns well with the results presented in this paper.

In the quantitative study of damage, a steel bar with a diameter of 20 mm and a length of 1 m is established. Five different defect scenarios are configured, each comprising a combination of corrosion lengths of 20 cm, 30 cm, and 40 cm and corrosion depths of 2 mm, 6 mm, and 10 mm, respectively. A torsional guided wave at a frequency of 50 kHz is selected to be excited at the left end of the steel bar and received at the right end. The wave transmitted through the damage defect is compared with the wave packet received under non-destructive conditions. By utilizing the root mean square error (RMSD) data-processing method [30], the damage index D is defined as the basis for determining the damage degree of the steel bar, as shown in Equation (2):

(2) $$D=\sqrt{\frac{\sum_{i=1}^{n}{\left(S_{i}-S_{0}\right)}^{2}}{n}}$$

where, *S*_i_ is the damage signal, *S*_0_ is the non-damage signal, and *n* is the sampling point.

**Figure 13:**
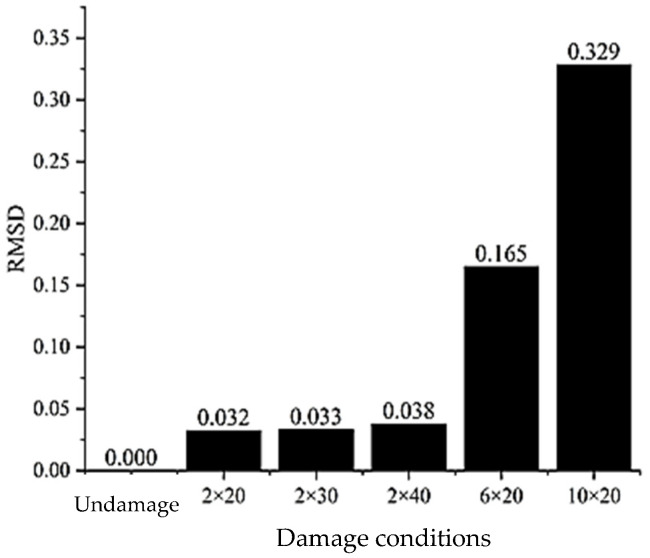
Damage index of a uniform defect.

As depicted in Figure 13, which illustrates a comparison of the damage index under different damage conditions, it is evident from the RMSD evaluation results that when the defect length remains constant, the damage index is highly sensitive to the defect depth, exhibiting a significant increase with the depth of the defect. This sensitivity arises from the profound impact of the depth on reflectivity. As observed in Figure 1b, the displacement of T (0, 1) varies significantly along the radial direction. Consequently, the absence of material in the radial direction significantly affects the propagation of guided wave energy, leading to more pronounced reflection signals from the defect. Thus, the greater the depth, the higher the damage index, with the sensitivity diminishing for smaller depths. This finding aligns with that of Yeung and Ng regarding crack depth monitoring in pipes using the SFEM method [16]. Moreover, under a certain defect depth, the damage index effectively identifies defects, albeit with only a slight increase as the defect length grows. In conclusion, the damage index serves as a reliable basis for determining the degree of damage in a steel bar.

**Figure 14:**
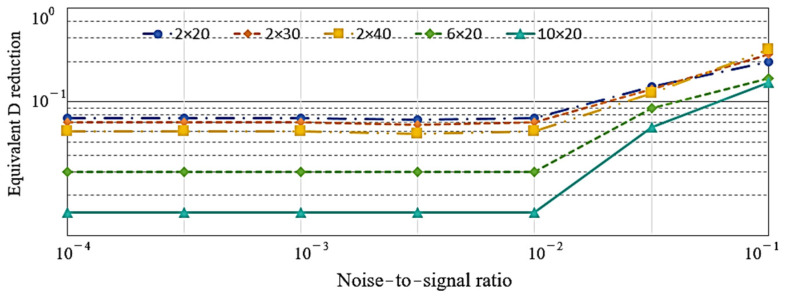
Influence of measurement noise and damage conditions on the equivalent D reduction.

In this study, the effects of measurement noise on the damage index were further investigated. Different noise levels were assumed, with the noise-to-signal ratio (NSR) ranging from 0.0001 to 0.1. Figure 14 illustrates the variations in equivalent D reduction (the ratio of the difference to the original value in the damage index) under different noise levels and damage conditions. The equivalent D reduction indicator exhibits a relatively slight increase for cases with NSRs < 0.01, but increases noticeably for cases with an NSR equaling 0.055. Meanwhile, the discrepancy of the predicted equivalent D reduction decreases as the severity of the damage increases. For example, in the case of NSR = 0.055, the predicted equivalent D reductions are 15.4%, 14.9%, 13.8%, 8.6%, and 6.3% for the damage conditions of 2 × 20, 2 × 30, 2 × 40, 6 × 20, and 10 × 20, respectively. Regarding measurement noise, the influences appear to be more pronounced in cases of slight damage compared to severe damage. However, this observation indicates that the damage index does not significantly decrease even when measurements are heavily noise-polluted. Therefore, the detection capability of the proposed damage index using the torsional mode of T (0, 1) remains evident in most cases in this particular example, particularly for cases with minor damage.

## 4. Conclusions

It is inferred from the waveform structure diagram of the guided wave that the torsional guided wave T (0, 1) exhibits greater sensitivity to detecting defects on the circular surface of the waveguide medium or even beneath the shallow surface compared to the commonly used longitudinal waves. This observation is effectively confirmed by numerical simulations. The simulation results reveal that when the depth of the groove defect on the circumferential surface is less than 50% of the diameter of the steel bar, and when the defect depth from the shallow surface to the circumferential surface is less than 25% of the diameter length of the steel bar, the defect reflectivity of the torsional wave surpasses that of the longitudinal wave. In fact, the maximum reflectivity of the torsional wave can be as much as twice that of the longitudinal wave, underscoring the superiority of the torsional guided wave in defect monitoring. These findings provide a theoretical underpinning for the practical application of torsional guided waves in monitoring scenarios. Moreover, a detailed investigation into the impact of changes in the defect’s size on monitoring reveals that the reflectivity of defects using torsional guided waves increases with the widening and deepening of the defect, allowing for characterization of the extent of structural damage through a fitted function. Additionally, numerical simulations for monitoring uniform damage defects in steel bars demonstrate that torsional guided waves exhibit greater sensitivity to detecting small defects compared to longitudinal guided waves. Furthermore, torsional guided waves can accurately locate and analyze defects, and evaluate the degree of damage in the steel bar by defining a damage index.

When a defect occurs on the surface or just beneath it, the reflectivity of the defect initially varies periodically with the extension of the axial length of the defect before stabilizing. This variability can complicate the assessment of the damage situation. Additionally, the damage index exhibits less sensitivity to changes in defect length compared to changes in depth. As a result, the influence of damage length on defect monitoring will be a primary focus in future research efforts.

The bare steel bar serves as the simplest and most fundamental component type, making it an ideal starting point for early research and discussions surrounding it. This groundwork will aid in laying the foundation for subsequent studies involving other types of components. The numerical simulation research conducted in this paper, based on T (0, 1), represents an initial step in comparison with research based on longitudinal wave. In the next phase, laboratory experiments and field applications will be undertaken to assess the suitability of this technology for damage monitoring. Notably, recent successful efforts by some researchers have involved exciting the T (0, 1) mode using PZT transducers, further supporting the feasibility of this approach.

## Figures and Tables

**Table 1:** Determination of defect location and length.

Conditions	First Wave Time/×10^−4^ s	Front-End Echo Time/×10^−4^ s	Starting Position of the Defect/m	Position Error/%	End Echo Time/×10^−4^ s	Time Difference/×10^−4^ s	Defect Length/m	Length Error/%
0.3–0.2–0.5	0.513	2.360	0.2990	0.34	3.580	1.220	0.198	1.24
0.6–0.2–0.2	0.504	4.196	0.5977	0.03	5.408	1.212	0.196	1.89
0.16–0.32–0.52	0.508	1.484	0.1580	1.24	3.432	1.948	0.315	1.45
0.38–0.46–0.16	0.513	2.856	0.3795	0.14	5.656	2.800	0.453	1.45

## Data Availability

Data are contained within the article.
